# Iatrogenic pseudoaneurysm rupture of the middle cerebral artery after an intracranial pressure monitor placement: a case report and literature review

**DOI:** 10.1007/s10072-023-06987-3

**Published:** 2023-08-10

**Authors:** Haibin Wang, Jinming Chen, Zhiqiang Gao, Zhaobin Zeng

**Affiliations:** Institute of Neurology, Department of Neurosurgery, The First Affiliated Hospital of Gannan Medical University, No. 23Qingnian Road, Zhanggong District, Ganzhou, 341000 Jiangxi China

**Keywords:** Intracranial pressure monitor, Iatrogenic pseudoaneurysm, Rupture, Resection

## Abstract

Thalamic hemorrhage (TH) is a devastating disease with a high mortality rate; however, no specific form of therapy has been proven to reduce mortality. Patients with hemorrhagic stroke undergo intracranial pressure (ICP) monitoring. However, cases involving pseudoaneurysms caused by ICP monitoring in patients with intracerebral hemorrhage have not been reported previously. Here, we report a case of pseudoaneurysm caused by an ICP monitor that was fitted due to hypertensive cerebral hemorrhage.

## Introduction

Thalamic hemorrhage (TH) accounts for 10 to 15% of cases involving intracerebral hemorrhage (ICH), thus resulting in high mortality and disability rates [1]. Minimally invasive puncture and drainage of a thalamic hematoma has been demonstrated to improve patient outcome [2]. The management guidelines for spontaneous ICH in the USA recommend the use of ICP monitoring for patients with ICH whose GCS score is less than 8, or the patient has hydrocephalus or intraventricular hemorrhage [3]. ICP monitoring is the basic form of management for patients with severe neurological conditions. External ventricular drain (EVD) is considered to be the gold standard [4]. Although it is considered to be safe, there are also complications related to infection and cerebral hemorrhage. Pseudoaneurysms caused by ICP monitoring placement are rare; only a few cases have been reported in the literature thus far. Here, we report a case of pseudoaneurysm of the terminal segment of the left middle cerebral artery caused by an ICP monitor which was resected successfully. In addition, we reviewed the published cases of pseudoaneurysms caused by ICP monitoring placement and the different treatment approaches used to treat these patients.

## Case report

**Fig. 1:**
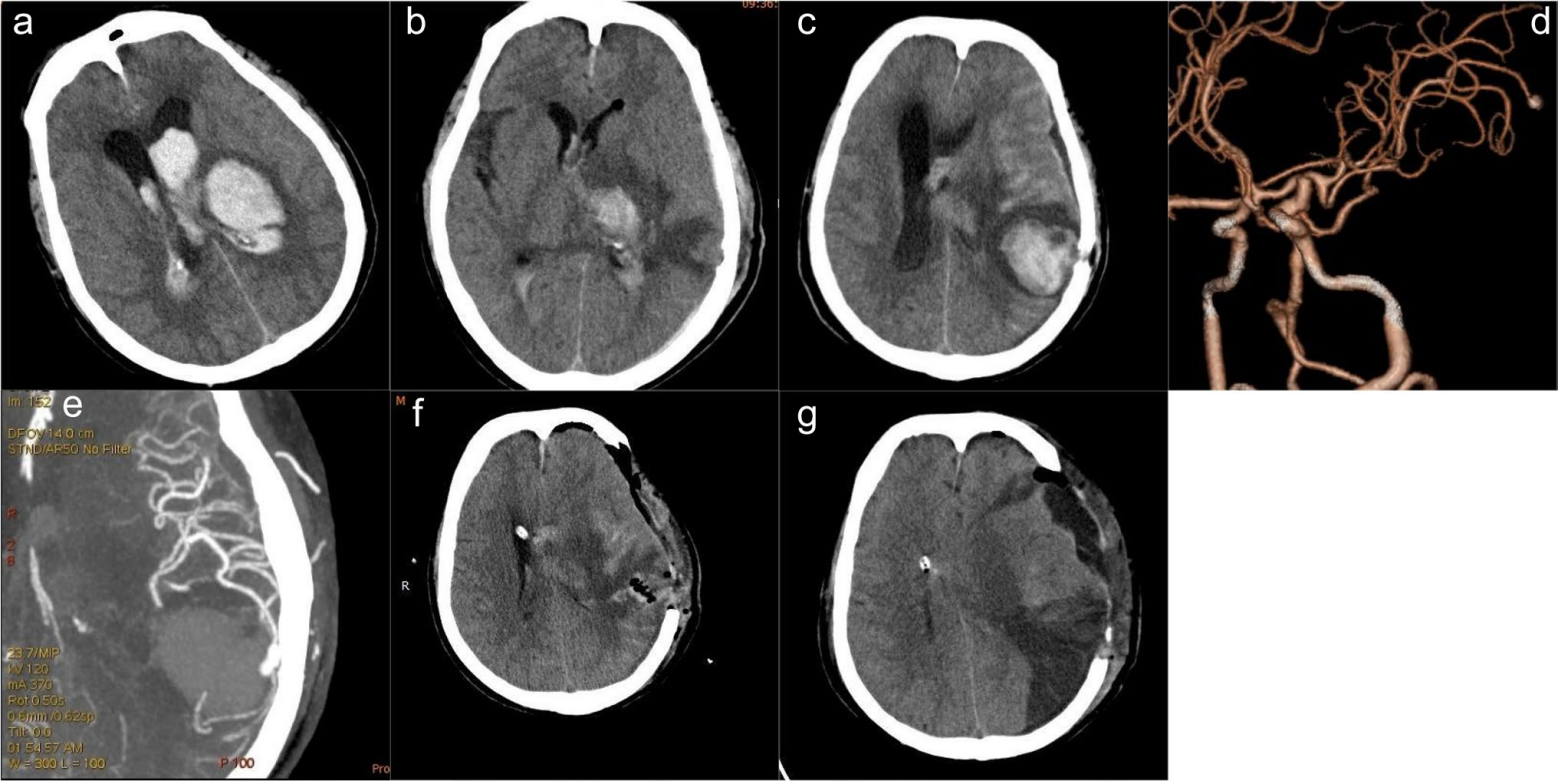
Neuroimaging acquired from our patient: **a** axial head computed tomography on initial patient presentation showing left thalamus hemorrhage, intraventricular extension, and hydrocephalus; **b** CT scan performed on the 6th postoperative day showing that the TH was almost eliminated; **c** CT scan performed on the 18th postoperative day showing a new intraparenchymal hemorrhage with 6mm of midline shift; (**d, e**) computed tomography angiography(CTA) showing a left distal middle cerebral artery pseudoaneurysm (red arrow); **f** CT scan performed on 8-h post-craniotomy showing that the hemorrhage had been evacuated completely and the bilateral ventricles had shrunk; **g** CT scan performed on the fourth day after the post-craniotomy showing left parietal lobe cerebral infarction

A 55-year-old male patient was taken to a local hospital with a coma, diagnosed with a TH, and was transferred to our hospital 8 h later. He had poorly controlled hypertension and was otherwise healthy. A neurological examination revealed that the right limb was paralyzed and that his Glasgow Coma Scale (GCS) was 8. A computed tomography (CT) scan revealed a left TH with intraventricular extension and acute hydrocephalus (Fig. 1a), with no increase in hematoma volume when compared with a previous CT carried out at the local hospital.

The patient underwent a left frontal EVD and minimally invasive puncture and drainage of left thalamic hematoma under general anesthesia. Due to the lack of an EVD-converting ICP device, an ICP sensor (Codman) was subsequently placed next to the drainage tube of the hematoma cavity. The sensor was inserted into the brain tissue at a depth of 1cm; no bleeding was observed during surgery. The patient was then returned to the neurosurgery intensive care unit (NSICU) for further treatment. A review of the head CT acquired on the first postoperative day showed that the drainage tubes were in the left ventricle and the hematoma cavity; liquefacient (5 ml of saline solution/20,000 U urokinase) was injected into the drainage tubes twice daily. On the 6th postoperative day, a routine head CT showed little residual hematoma in the ventricle and left intraparenchymal (Fig. 1b). The patient was in a stable condition; therefore, the drainage tubes and sensor were removed without complications. On the 18th postoperative day, he developed dilation of the left pupil; his GCS was 5. An urgent head CT revealed a new left parietal hemorrhage (Fig. 1c), and head CTA revealed a left parietal pseudoaneurysm arising from a distal branch of the left middle cerebral artery (MCA) underneath the burr hole (Fig. 1d and e).

Head CT presented with midline shift and hydrocephalus. Consequently, the patient was sent to the operating room for a left-sided decompressive craniectomy. We evacuated the hematoma, ligated the parent artery, and resected the pseudoaneurysm. An EVD was placed in the right frontal region. An 8-h post-craniotomy head CT showed that the hematoma had been evacuated completely and that the bilateral ventricles had shrunk (Fig. 1f). Unfortunately, on the fourth day post-craniotomy, he had a poor response. The head CT showed ischemic infarction in the left parietal lobe (Fig. 1g). He was transferred to another medical facility for further treatment. The patient is now being followed up as an outpatient, and his modified Rankin Scale score at 6 months post-surgery was 4.

## Discussion

Intracranial pseudoaneurysms account for 1% of all intracranial aneurysms and are associated with a mortality rate of up to 20% [5]. The iatrogenic formation of intracranial pseudoaneurysm has been associated with certain types of neurosurgical operations, such as transsphenoidal surgery, external ventricular drain insertion, and other procedures. Numerous clinical studies have shown that minimally invasive puncture and drainage for the evacuation of ICH are a safe and effective surgical procedure for the treatment of hypertensive thalamic hemorrhage, thus reducing the risk of vascular injury during the procedure. Since the vessels surrounding the channel are pushed apart by a smooth rod during the puncture of the drainage tube into the hematoma cavity [2], there are no reports in the literature related to the pseudoaneurysm. However, a penetrating intracerebral parenchymal surgery can lead to arterial injury, and full-layer arterial injury leads to the formation of false aneurysms, which are prone to delayed postoperative bleeding at the surgical site. In our case, pseudoaneurysm appeared in the same location as the sensor placement. Therefore, we believed that the pseudoaneurysm originated from the implantation site of the monitor.

Only three cases of iatrogenic intracranial pseudoaneurysms caused by ICP monitoring have been reported in the literature (Table 1). Currently, the management of a ruptured pseudoaneurysm includes microsurgery [6], embolization [7], and conservative treatment. The choice of procedure depends on the location of the pseudoaneurysm and the patient’s neurological status following the rupture. Of the reported cases, one case of left distal M4 frontal branch pseudoaneurysm was successfully embolized; the others were surgically removed due to their superficial location. Similarly, in our case, the pseudoaneurysm was located in the cortex of the left middle cerebral artery and head CT presented with midline shift preoperatively. Therefore, we chose emergency decompressive craniectomy to ligate the feeding artery and resect the pseudoaneurysm; then, we evacuated the hematoma. Unfortunately, the patient developed an acute cerebral infarction in the left parietal lobe on the fourth postoperative day. We inferred from the scope of the infarction that it may have been caused by intraoperative distal MCA closure.

**Table 1:** Reported cases of iatrogenic pseudoaneurysms caused by intracranial pressure monitor

Case	Patient age	Gender	Location	Cause	Diagnosis prompted by	Treatment	Outcome	Reference
1	30	Male	Fusiform aneurysm of a branch of the right ACA	Insertion of an ICP monitor	A routine CT scan obtained 25 days post-injury	Surgical resection	NA	[8]
2	27	Male	Fusiform pseudoaneurysm of a frontal branch of the distal MCA	Insertion of an ICP monitor	Dilation of the left pupil and right gaze,17 days after the procedure	Embolization with n-butyl cyanoacrylate	Speak in short phrases, feed himself with the use of his right arm	[9]
3	30	Male	An anterior branch of the STA	Insertion of an ICP monitor	Follow-up with a mass prompted CTA at 6 weeks	Ligated the feeding artery and resection	Recovered well	[10]
4	55	Male	Fusiform pseudoaneurysm of a branch of the distal MCA	Insertion of an ICP monitor	Dilation of the left pupil 18 days after the procedure	Ligated the feeding artery and resection	A modified Rankin Scale score of 4	Present case

*ACA*, anterior cerebral artery; *STA*, superficial temporal artery; *MCA*, middle cerebral artery; *ICP*, intracranial pressure; *NA*, not available; *CTA*, computed tomography angiography

ICP probes are usually placed in the right frontal region. However, the placement can be modified depending on known or suspected pressure gradients across intracranial compartments [11]. In this case, the pressure gradient in the left temporal region was significantly elevated; therefore, we placed the ICP probe next to the drainage tube. Although ICP monitoring is well established, its manipulation of brain tissue may lead to vessel wall injury, thus resulting in the formation of a pseudoaneurysm. These cases highlight the need for neurosurgeons to be cautious and avoid the vascular distribution area as far as possible when performing invasive procedures. ICP monitoring-associated pseudoaneurysms are rare; they also have a high propensity for rupture. Therefore, it is important to treat these lesions early in order to prevent deterioration.

## Abbreviations

TH: Thalamic hemorrhage
ICH: Intracerebral hemorrhage
EVD: External ventricular drainage
MCA: Middle cerebral artery
CT: Computed tomography
NSICU: Neurosurgery intensive care unit
CTA: Computed tomography angiography
ICP: Intracranial pressure
GCS: Glasgow Coma Scale
mRS: Modified Rankin Scale score
